# Postoperative improvement in leg length discrepancy in adolescent idiopathic scoliosis differs between right and left legs^*^

**DOI:** 10.1016/j.xnsj.2022.100114

**Published:** 2022-03-21

**Authors:** Yusuke Sakai, Shota Takenaka, Takahiro Makino, Takashi Kaito

**Affiliations:** Department of Orthopaedic Surgery, Osaka University Graduate School of Medicine, 2-2 Yamadaoka, Suita, Osaka, 565-0871, Japan

**Keywords:** Leg length discrepancy, Adolescent idiopathic scoliosis, Posterior corrective surgery, Postoperative improvement

## Abstract

**Background:**

In adolescent idiopathic scoliosis (AIS) patients, leg length discrepancies (LLDs) often occur to compensate for scoliosis. However, there have been no reports on the LLD changes after corrective surgery for AIS. This study aimed to clarify the difference of LLD changes after corrective surgery for AIS by classifying LLD based on the shortened side.

**Methods:**

We analyzed preoperative and postoperative radiographs of 94 consecutive AIS patients who underwent posterior corrective surgery between 2012 and 2018. The patients enrolled were divided into three groups according to the presence of preoperative LLD of more than 5 mm and the LLD side: the left leg shortened group (L group), the non-LLD group (N group), and the right leg shortened group (R group). The three groups were compared with regard to age, sex, Lenke classification, Risser grade, fused levels, and radiographic parameters before surgery and at 6-month follow-up (thoracic Cobb angle, lumbar Cobb angle, L4 tilt, coronal balance, T1 tilt, and LLD).

**Results:**

The L, N, and R groups included 23 (24%), 60 (64%), and 11 patients (12%), respectively. The demographics and radiographic parameters were not significantly different among the groups except for preoperative L4 tilt. In the L group only, the LLD decreased from 7.9 ± 2.2 mm to 5.7 ± 3.7 mm (*p* = 0.002) after surgery. In contrast, the LLD in the N and R groups did not change significantly.

**Conclusions:**

The postoperative improvement of LLD in AIS patients differed between the left and right sides. Different pathologies may contribute to the LLD on the left and right sides.

## Introduction

Leg length discrepancy (LLD) is often observed in standing posterior-anterior radiographs in patients with adolescent idiopathic scoliosis (AIS). There are two types of LLD. One is called structural LLD, which involves the actual shortening of a leg, and the other is called functional LLD, which does not involve the actual shortening of a leg and is frequently observed in AIS [1]. Structural LLD causes pelvic obliquity in the frontal plane, and it leads to lumbar scoliosis with convexity towards the shorter leg [2,3]. Meanwhile, the functional LLD occurs to compensate for scoliosis, often with pelvic rotation [4], [5], [6]. Thus, LLD and scoliosis can influence each other.

A recent study on whole-body radiography in the upright position has shown that most LLDs in AIS patients are functional LLDs [7]. Therefore, surgical correction of the coronal curve in AIS should lead to a decrease in LLD; however, there have been no reports on the postoperative changes in LLD.

The purpose of this study was to investigate the postoperative changes in LLD in AIS and to clarify the difference in the improvement between LLD on the right and left sides.

## Materials and methods

This study was reviewed and approved by our institution's ethics committee. Patients were given the opportunity to opt out of the study.

**Figure 1:**
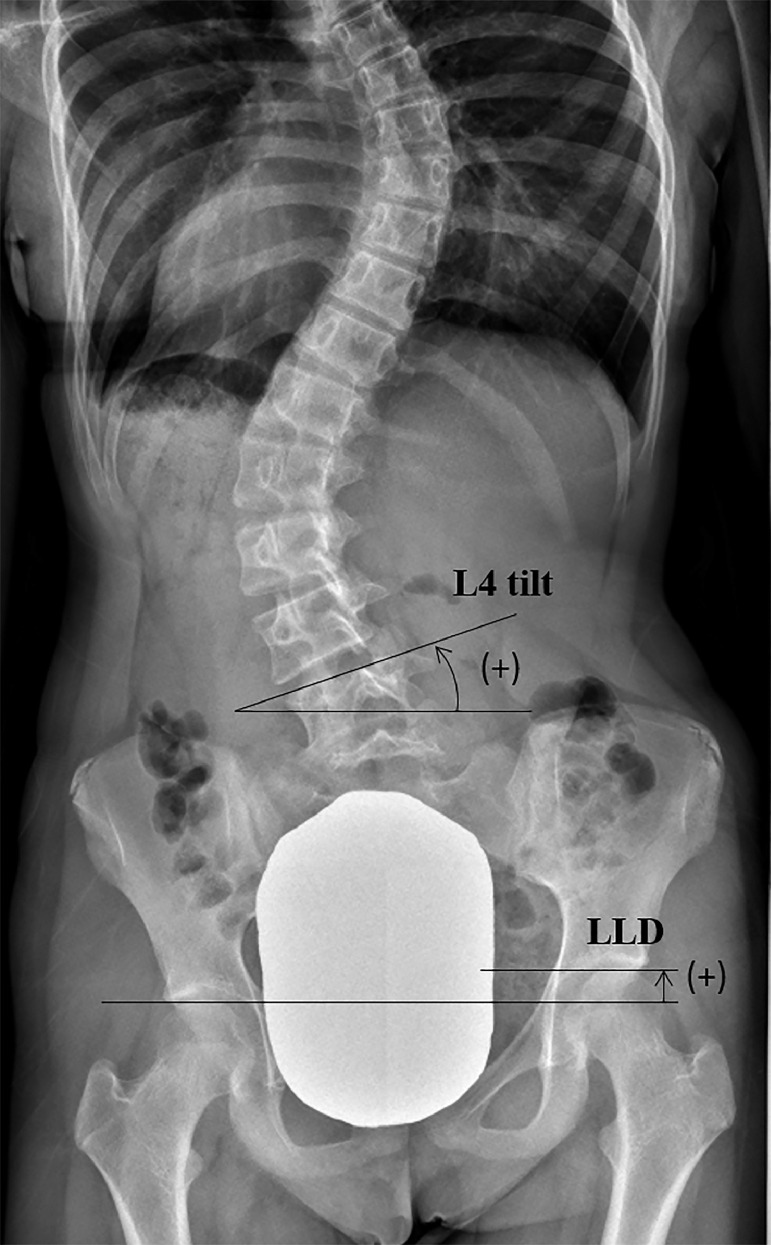
Leg length discrepancy (LLD) was defined as the height difference between the tops of the bilateral femoral heads; a positive value for left-side-down position.

We retrospectively reviewed 94 consecutive AIS patients (90 women and 4 men; mean age of 16.8 years; irrespective of curve type) who received posterior corrective surgery between 2012 and 2018. LLD was defined as a >5 mm height difference between the tops of the bilateral femoral heads on whole spine standing posterior-anterior radiographs (a positive value for left-side-down position; Fig. 1). The rationale for the 5 mm threshold is that LLD of 5 mm is the minimum magnitude that causes subjective symptoms such as low back pain [8,9]. One patient who used orthotics preoperatively, that is, a patient with known structural LLD, was excluded from the analyses.

Patients were divided into three groups according to the existence of LLD and the side of the shortened leg (L group, short left leg; N group, no LLD; R group, short right leg). In five cases with lumbar curve convex to the right, LLD was evaluated by inverting the left and right sides. The three groups were compared in terms of age at surgery, sex, Lenke classification, Risser grade (classified into 3 categories of 0–3, 4, and 5), upper instrumented vertebra (UIV; classified into 3 categories of T1–3, T4–6, and T7 or below), lower instrumented vertebra (LIV), number of fused levels, and the following radiographic parameters: Cobb angles of thoracic (main thoracic) and lumbar (thoracolumbar) curves, L4 tilt, coronal balance (distance between C7 plumb line and the central sacral vertical line), T1 tilt, and changes in LLD at 6 months after surgery. A positive value for L4 tilt and T1 tilt was defined as inclination to the left, and a positive value of the coronal balance was defined as a shift to the right.

In addition to the aforementioned comparison among the three groups, we investigated the possible confounding relationship between the preoperative LLD and the other preoperative radiographic parameters. We also classified the L and R groups into two subgroups each to investigate the preoperative factors related to whether the LLD was reduced to 5 mm or less postoperatively. The two subgroups were compared in terms of age at surgery, Lenke classification, Risser grade, and preoperative radiographic parameters to investigate the predictors of postoperative LLD improvement.

### Reproducibility evaluation

To evaluate intraobserver and interobserver reliability, 30 patients were randomly selected to undergo measurements of preoperative LLDs in duplicate, with a 2-week interval, by an investigator and a second observer. Both observers were orthopedic spine surgeons blinded to the subject data. The reproducibility of the LLD was evaluated using intraclass correlation coefficients. The intraobserver and interobserver reliabilities were almost perfect [10] (Table 1).

**Table 1:** Reliability test for leg length discrepancy.

	ICC (95% CI)	RMSE
Intraobserver	0.995 (0.990–0.998)	0.5 mm
Interobserver	0.995 (0.989–0.997)	0.6 mm

ICC, intraclass correlation coefficients; CI, confidence interval; RMSE, root mean squared error.

### Surgical procedures

All patients underwent posterior corrective surgery with hybrid constructs or all-pedicle screw constructs. After the placement of hooks and screws, a rod contoured to the thoracic kyphosis or the lumbar lordosis was placed and rotated 90° for scoliosis correction, and kyphosis or lordosis creation. Scoliosis was further corrected with *in situ* benders. The other side rod was placed, and segmental compression and distraction were performed. Direct vertebral derotation via pedicle screws placed on both sides was additionally performed in patients with all-pedicle screw constructs.

### Statistical analysis

SPSS statistical software (v. 21.0; IBM Corp., Armonk, NY, USA) was used for all statistical analyses. Prior power analysis was conducted to calculate the minimum sample size necessary to detect a 3.0 mm difference of postoperative LLD changes as a paired t-test (calculated with assumed standard deviation = 3.5 mm, alpha = 0.05, power = 0.8). The analysis indicated a required sample size of 11 patients per group. A *p*-value <0.05 was considered significant in all analyses. Differences between the three groups were analyzed by Fisher's exact test for nominal scales and Kruskal-Wallis test for ratio scales. When there was a significant difference, the Bonferroni post hoc test was performed using Fisher's exact test for nominal scales and the Mann-Whitney U test for ratio scales to compare two groups with adjusted *p*-value (*p*-value multiplied by 3). LLD change before and after surgery in each group was analyzed by the Wilcoxon signed-rank test. Pearson correlation analysis was used to assess correlations among parametric variables. The correlation coefficient was interpreted as follows: > 0.9, very high correlation; 0.7–0.9, high correlation; 0.4–0.7, moderate correlation; 0.2–0.4, low correlation; < 0.2, no correlation [11].

## Results

The L group, N group, and R group included 23 patients (24%), 60 patients (64%), and 11 patients (12%), respectively. There were no significant differences between groups in age at surgery, sex, Lenke type, Risser grade, UIV, LIV, and fused levels (Table 2). In the radiographic parameters, preoperative L4 tilt was significantly different among the groups (*p* = 0.025, Kruskal-Wallis test). The L group had larger L4 tilt than the R group (12 ± 10 degrees vs. -3 ± 18 degrees, with adjusted *p* = 0.045 by Bonferroni post hoc test). There was no difference in thoracic Cobb angle, lumbar Cobb angle, coronal balance, and T1 tilt between the three groups both preoperatively and postoperatively (Table 3).

**Table 2:** Comparison of the demographic features of groups.

	L group	N group	R group	*p*-value
Number	23 (24%)	60 (63%)	11 (12%)	
Age (range), years	15.4 ± 3.1 (12–23)	17.4 ± 5.2 (10–29)	17.4 ± 5.8 (12–34)	0.117 K
Sex, male/female	1/22	3/57	0/11	1.000 F
Lenke type				0.344 F
** **1	13	33	5	
** **2	3	12	4	
** **3	0	2	0	
** **4	1	0	1	
** **5	5	9	0	
** **6	1	4	1	
Lenke lumbar modifier				0.040 F^,^*
** **A	4	23	7	
** **B	4	16	1	
** **C	15	21	3	
Risser grade				0.293 F
** **0–3	5	6	1	
** **4	12	24	5	
** **5	6	30	5	
UIV				0.505 F
** **T1–3	3	13	3	
** **T4–6	15	39	7	
** **T7 or below	5	8	0	
LIV				0.362 F
** **T12	4	13	0	
** **L1	3	15	2	
** **L2	4	14	5	
** **L3	12	18	4	
Fused levels (range)	9.8 ± 2.4 (6–14)	9.9 ± 1.8 (6–13)	11.3 ± 1.5 (6–14)	0.108 K

UIV, upper instrumented vertebra; LIV, lower instrumented vertebra.

K Kruskal-Wallis test was used for continuous variables.

F Fisher's exact test was used for categorical variables.

⁎ No significant difference was shown by Bonferroni post hoc test. Adjusted *p*-value was 0.093 between the L group and the R group.

**Table 3:** Comparison of radiographic parameters.

	L group(n = 23)	N group(n = 60)	R group(n = 11)	*p*-value
Preoperative				
** **Thoracic Cobb angle, degree	51 ± 16 (22–80)	53 ± 15 (12–99)	60 ± 17 (45–102)	0.508
** **Lumbar Cobb angle, degree	42 ± 12 (20–73)	39 ± 15 (14–73)	34 ± 15 (14–60)	0.261
** **L4 tilt, degree	12 ± 10 (-17–27)	8 ± 13 (-20–34)	-3 ± 18 (-27–24)	0.025*
** **Coronal balance, mm	-5 ± 15 (-29–28)	-5 ± 17 (-54–29)	2 ± 17 (-25–21)	0.357
** **T1 tilt, degree	-1 ± 6 (-18–9)	1 ± 7 (-17–24)	-1 ± 7 (-11–14)	0.602
** **LLD, mm	7.9 ± 2.2 (5.1–12.7)	0.8 ± 2.7 (-4.8–4.5)	-7.8 ± 3.5 (-16.5–-5.1)	< 0.001
Postoperative				
** **Thoracic Cobb angle, degree	20 ± 5 (12–30)	21 ± 8 (7–47)	22 ± 10 (9–42)	0.967
** **Lumbar Cobb angle, degree	16 ± 4 (7–24)	16 ± 9 (1–39)	14 ± 11 (0–33)	0.602
** **L4 tilt, degree	6 ± 5 (-11–13)	5 ± 7 (-13–17)	2 ± 11 (-15–18)	0.523
** **Coronal balance, mm	-10 ± 13 (-36–10)	-12 ± 14 (-46–23)	-15 ± 11 (-34–0)	0.434
** **T1 tilt, degree	-6 ± 6 (-20–8)	-6 ± 7 (-19–13)	-5 ± 6 (-12–7)	0.938
** **LLD, mm	5.7 ± 3.7 (0.7–13.4)	-0.2 ± 3.3 (-8.2–6.9)	-6.2 ± 5.1 (-12.9–7.1)	< 0.001

LLD, leg length discrepancy.

Kruskal-Wallis test was used for all variables.

⁎ Adjusted *p*-value by Bonferroni post hoc test using Mann-Whitney U test was 0.045 between the L group and the R group.

**Figure 2:**
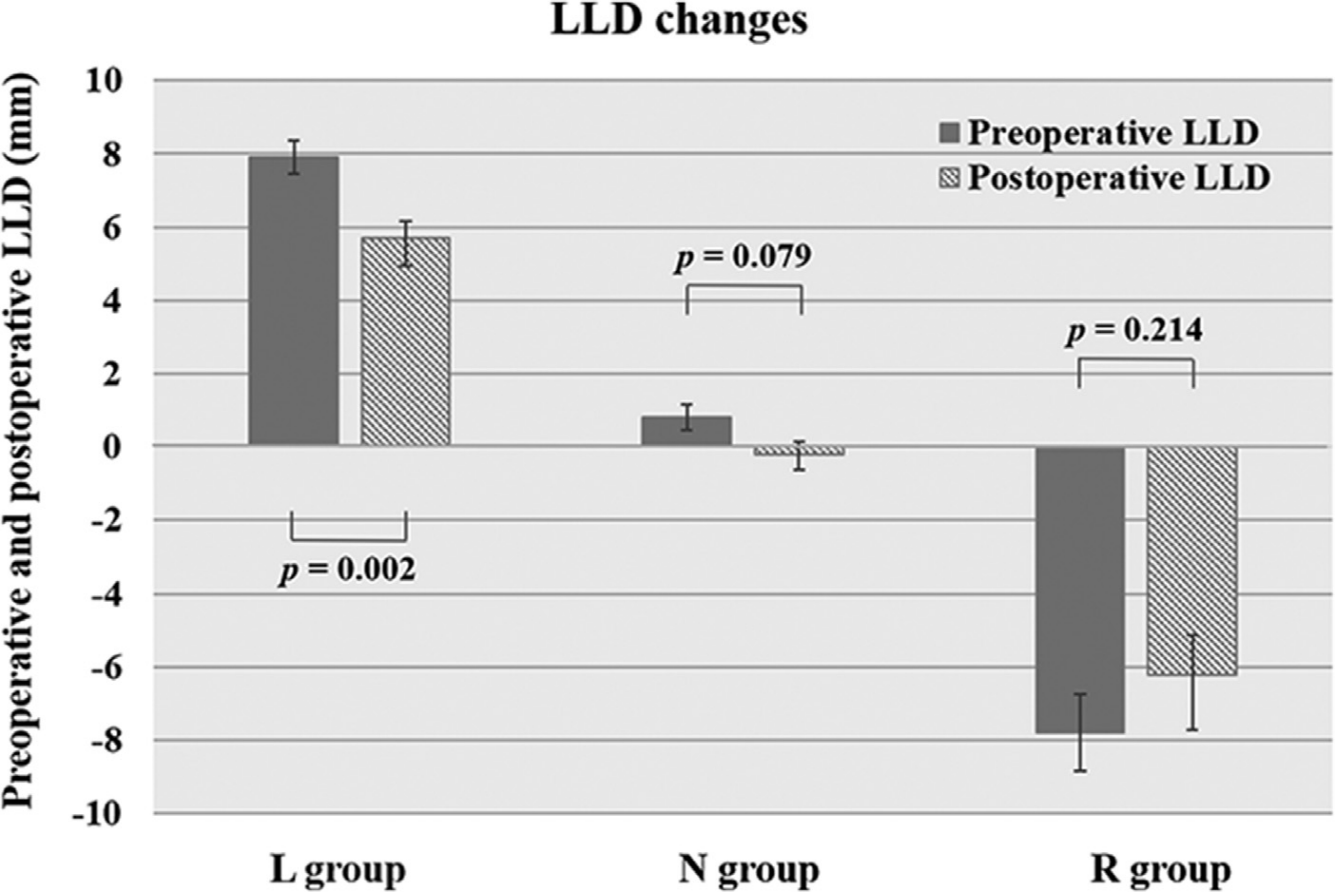
Comparison of the leg length discrepancy (LLD) changes from preoperative to 6 months postoperative in each group. The three groups L, N and R represent the presence of preoperative LLD of more than 5 mm and the LLD side: the left leg shortened group (L group), the non-LLD group (N group), and the right leg shortened group (R group). The values are expressed as the mean ± standard error.

Only in the L group, the LLD decreased from 7.9 ± 2.2 mm preoperatively to 5.7 ± 3.7 mm postoperatively (*p* = 0.002, Wilcoxon signed-rank test). In contrast, LLD in the N group and the R group did not change (the N group: from 0.8 ± 2.7 mm to -0.2 ± 3.3 mm, *p* = 0.079; the R group: from -7.8 ± 3.5 mm to -6.2 ± 5.1 mm, *p* = 0.214, Fig. 2).

The results of Pearson correlation analysis between the preoperative LLD and the other preoperative radiographic parameters are shown in Table 4. The results showed that the preoperative LLD was weakly correlated with the preoperative L4 tilt (*r* = 0.25, *p* = 0.014), but not with the other parameters.

**Table 4:** Pearson correlation analysis between preoperative LLD and other radiographic parameters.

	LLD
L4 tilt	0.25 (*p* = 0.014)
Lumbar Cobb angle	0.10 (*p* = 0.345)
Thoracic Cobb angle	-0.14 (*p* = 0.189)
T1 tilt	-0.04 (*p* = 0.723)
Coronal balance	-0.06 (*p* = 0.572)

LLD, leg length discrepancy.

In the L group, 11 of 23 cases achieved a reduction of LLD to 5 mm or less after surgery. In contrast, in the R group, LLD decreased in only 1 of 11 cases. Comparing the subgroup with and without LLD decrease in the L group, the subgroup with LLD decrease had smaller preoperative LLD than the subgroup without LLD decrease (6.8 ± 2.0 mm vs. 8.9 ± 2.0 mm, *p* = 0.019). No significant differences were found in age at surgery, Lenke classification, Risser grade, and the other preoperative radiographic parameters (Table 5).

**Table 5:** Comparison between the subgroups with and without LLD decrease in the L group.

	With LLD decrease	Without LLD decrease	*p*-value
Number	11	12	
Age (range), years	14.7 ± 3.0 (12–23)	16.0 ± 3.1 (13–21)	0.344 M
Lenke type			0.216 F
** **1	5	8	
** **2	3	0	
** **3	0	0	
** **4	0	1	
** **5	2	3	
** **6	1	0	
Lenke lumbar modifier			0.829 F
** **A	1	3	
** **B	2	2	
** **C	8	7	
Risser grade			0.746 F
** **0–3	3	2	
** **4	6	6	
** **5	2	4	
Preoperative radiographic parameters			
** **Thoracic Cobb angle, degree	52 ± 15 (22–72)	49 ± 17 (22–80)	0.600 M
** **Lumbar Cobb angle, degree	45 ± 13 (26–73)	39 ± 11 (20–60)	0.195 M
** **L4 tilt, degree	14 ± 11 (-3–26)	9 ± 11 (-17–27)	0.735 M
** **Coronal balance, mm	-2 ± 15 (-29–16)	-7 ± 15 (-28–28)	0.295 M
** **T1 tilt, degree	-2 ± 8 (-18–9)	1 ± 4 (-7–9)	0.516 M
** **LLD, mm	6.8 ± 2.0 (5.1–11.7)	8.9 ± 2.0 (5.1–12.7)	0.019 M

LLD, leg length discrepancy.

M Mann-Whitney U test was used for continuous variables.

F Fisher's exact test was used for categorical variables.

## Discussion

In this study, we investigated changes in LLD after corrective surgery for scoliosis. The results showed that LLD did not decrease after corrective surgery in the group with right leg shortening. Our results suggest a different pathology between left and right leg shortening. The preoperative LLD was weakly correlated with the preoperative L4 tilt, but not with the other parameters.

LLD can be subdivided into structural LLD with bone shortening and functional LLD caused by compensatory mechanisms for scoliosis [1]. Scoliosis caused by structural LLD improves completely or partially when the LLD is treated [3,12]. However, it remains unknown whether the functional LLD caused by scoliosis improves after scoliosis is surgically corrected.

The majority of LLD in AIS is reported to be functional [7]. If all functional LLD is caused by compensatory mechanisms for scoliosis, the leg shortening in AIS patients should be observed in the left leg because the majority of lumbar curve is left convex. However, in this study, a fair number of patients with shortened right legs were observed. Moreover, LLD did not improve after surgery in almost all patients with shortened right leg and in half of patients with shortened left leg. These results suggest that various factors other than scoliosis are involved in the development of functional LLD, including pelvic/trunk rotation and pelvic asymmetry [13].

A relationship between LLD and lumbar curve has been shown in some reports. Sekiya et al. examined whole-body radiography in 82 AIS patients using the EOS imaging system (Biospace Imaging, Paris, France), and reported that functional LLD was significantly correlated with the lumbar Cobb angle with a moderate correlation coefficient [7]. In contrast, Cho et al. analyzed 303 AIS patients’ whole spine radiographs and reported that there was no direct correlation between lumbar curve and LLD [14]. In this study as well, there was no correlation between the lumbar Cobb angle and LLD. However, the fact that L4 tilt was correlated with LLD suggests that scoliosis may have some influence on LLD.

There have been few reports on the correlation between LLD and thoracic curve. Sekiya et al. reported that there was no correlation between thoracic curve and LLD [7]. In this study, the preoperative thoracic Cobb angle was comparable between the groups. The effect of the thoracic curve on LLD might be weakened by the intervening lumbar curve between the thoracic curve and pelvis.

LLD is not directly caused by the curve size or the Lenke type, but may be affected by multiple factors such as pelvic/trunk rotation and pelvic asymmetry, in addition to the effect of L4 tilt. Ploumis et al. examined the radiographical changes of 73 non-operative AIS patients with an average follow-up of 2.8 years and found no significant change in LLD but significant increases in scoliotic and pelvic deformity parameters [15]. These results suggest that LLD is marshalled as the initial compensatory mechanism for scoliosis, but progressive pelvic asymmetry appears to compensate for the vertebral tilt of the lower lumbar spine with the progression of scoliosis.

Postoperative changes in pelvic obliquity were reported by Chen et al [13]. They described how pelvic obliquity could improve following the correction of scoliosis, but did not improve in the presence of structural problems such as pelvic hypoplasia. They also mentioned that intraoperative coronal balancing should consider the pelvic obliquity in patients with structural problems, particularly when fusion is extended to the lumbar spine. We think that these considerations regarding pelvic obliquity can be applied to LLD as well. Preoperative consideration should be given to whether correction of scoliosis will improve LLD or not. Although the mechanism of postoperative LLD improvement has not been completely clarified, our results suggest that right-shortened LLD is difficult to treat successfully.

This study has several limitations. First, we collected the data retrospectively, and whole leg radiographs were not available in all cases. However, the majority of LLD in AIS is functional and the risk of mixing structural LLD should be relatively small. Prospective studies combining whole leg and whole spine radiographs will strengthen the evidence in the future. Second, only posterior-anterior radiographs were evaluated in this study. Therefore, the rotation of the pelvis, which is also a factor that can affect LLD, was not evaluated. However, LLD is defined in posterior-anterior radiograph, and the rotation of the pelvis is considered to have a smaller impact than the coronal inclination of the pelvis. Third, the number of patients with right leg shortening was too small to make conclusions about the postoperative changes. Although these limitations are important, to the best of our knowledge, this is the first study to investigate LLD improvement after surgery in AIS patients.

## Conclusions

The postoperative improvement of LLD in AIS patients was limited to the patients with left leg shortening. This result suggests the existence of different mechanisms between right and left leg shortening. Although the etiology of right leg shortening remains unknown, the results of this study provided hints for considering the etiology and potential for changes of LLD.

## Declaration of Competing Interest

The authors declare that they have no known competing financial interests or personal relationships that could have appeared to influence the work reported in this paper.

## Appendix Supplementary materials

Image, application 1

Image, application 2

Image, application 3

Image, application 4

Image, application 5
