# Supplementary material for: Postoperative improvement in leg length discrepancy in adolescent idiopathic scoliosis differs between right and left legs
Source: N Am Spine Soc J. 2022 Mar 21;10:100114. doi: 10.1016/j.xnsj.2022.100114 (PMC9027341; doi:10.1016/j.xnsj.2022.100114)
Supplement: Supplementary file 2 [file mmc2.pdf]

ICMJE INTERNATIONAL COMMITTEE *of* MEDICAL JOURNAL EDITORS

ICMJE Form for Disclosure of Potential Conflicts of Interest

Section 1. Identifying Information

1. Given Name (First Name)
Takahiro

2. Surname (Last Name)
Makino

3. Date (use drop-down menu)
08-February-2022

4. Are you the corresponding author? ☐ Yes ☑ No

Corresponding Author's Name
Takashi Kaito

5. Manuscript Title
Postoperative Improvement in Leg Length Discrepancy in Adolescent Idiopathic Scoliosis Differs Between Right and Left Legs

6. Manuscript Identifying Number (if you know it)

Section 2. Relationships Pertaining to the Submitted Manuscript

Did you or your institution at any time receive payment or services from a third party for any aspect of the submitted work (including but not limited to grants, data monitoring board, study design, manuscript preparation, statistical analysis, etc…)?

Complete each row by checking "No" or providing the requested information. If you have more than one relationship click the "Add" button to add a row. Excess rows can be removed by clicking the "X" button.

Relationships Pertaining to Submitted Manuscript

Type	No	Money Paid to You	Money to Your Institution*	Name of Entity	Dollar Amount**	
1. Grant	☑	☐	☐			× ADD
2. Consulting fee or honorarium	☑	☐	☐			× ADD
3. Support for travel to meetings for the study or other purposes	☑	☐	☐			× ADD
4. Fees for participation in review activities such as data monitoring boards, statistical analysis, end point committees, and the like	☑	☐	☐			× ADD
5. Payment for writing or reviewing the manuscript	☑	☐	☐			× ADD
6. Provision of writing assistance, medicines, equipment, or administrative support	☑	☐	☐			×

ICMJE INTERNATIONAL COMMITTEE of MEDICAL JOURNAL EDITORS

ICMJE Form for Disclosure of Potential Conflicts of Interest

Relationships Pertaining to Submitted Manuscript

Type	No	Money Paid to You	Money to Your Institution*	Name of Entity	Dollar Amount**	
						ADD
7. Other	☑	☐	☐			×
						ADD

* This means money that your institution received for your efforts on this study.
** Dollar amount, number of shares, and/or percentage of ownership.

Section 3. Financial Relationships (Universal Disclosure)

Place a check in the appropriate boxes in the table to indicate whether you have financial relationships (regardless of amount of compensation) with entities as described in the instructions. Use one line for each entity; add as many lines as you need by clicking the "Add +" box. You should report relationships that were present during the 36 months prior to submission.

Complete each row by checking "No" or providing the requested information. If you have more than one relationship click the "Add" button to add a row. Excess rows can be removed by clicking the "X" button.

Relevant financial activities outside the submitted work

Type of Relationship (in alphabetical order)	No	Money Paid to You	Money to Your Institution*	Entity	Dollar Amount**	
1. Physician-Owned Distributorship	☑	☐	☐			×
						ADD
2. Royalties	☑	☐	☐			×
						ADD
3. Stock Ownership	☑	☐	☐			×
						ADD
4. Private Investments	☑	☐	☐			×
						ADD
5. Consulting	☑	☐	☐			×
						ADD
6. Speaking and/or Teaching Arrangements	☑	☐	☐			×
						ADD
7. Trips/Travel	☑	☐	☐			×

ICMJE Form for Disclosure of Potential Conflicts of Interest

Relevant financial activities outside the submitted work

Type of Relationship (in alphabetical order)	No	Money Paid to You	Money to Your Institution*	Entity	Dollar Amount**	
						ADD
8. Board of Directors	☑	☐	☐			×
						ADD
9. Scientific Advisory Board/Other Office	☑	☐	☐			×
						ADD
10. Endowments	☑	☐	☐			×
						ADD
11. Research Support (Investigator Salary, Staff/Materials)^	☑	☐	☐			×
						ADD
12. Grants	☑	☐	☐			×
						ADD
13. Fellowship Support	☑	☐	☐			×
						ADD

* This means money that your institution received for your efforts.
** Dollar amount, number of shares and/or percentage of ownership.
^ Indicate whether amount received was towards investigator salary and/or staff/materials.

Section 4. Other relationships

Authors should disclose other relationships, which would reasonably be judged to have a direct relationship to the topic of the activity. These relationships that could reasonably be judged by an observer to be related to the topic should be disclosed through general disclosure (ie, estimated dollar amounts are encouraged but not required).

☑ No other relationships/conditions/circumstances that present a potential conflict of interest

☐ Yes, the following relationships/conditions/circumstances are present (explain below):

At the time of manuscript acceptance, journals may ask authors to confirm and, if necessary, update their disclosure statements. On occasion, journals may ask authors to disclose further information about reported relationships.

Hide All Table Rows Checked 'No'   SAVE
